# Optically Trapped Bacteria Pairs Reveal Discrete Motile Response to Control Aggregation upon Cell–Cell Approach

**DOI:** 10.1007/s00284-014-0641-5

**Published:** 2014-06-26

**Authors:** Maria Dienerowitz, Laura V. Cowan, Graham M. Gibson, Rebecca Hay, Miles J. Padgett, Vernon R. Phoenix

**Affiliations:** 1 SUPA, School of Physics and Astronomy, University of Glasgow, Glasgow, G12 8QQ UK; 2 School of Geographical and Earth Sciences, University of Glasgow, Glasgow, G12 8QQ UK

## Abstract

Aggregation of bacteria plays a key role in the formation of many biofilms. The critical first step is cell–cell approach, and yet the ability of bacteria to control the likelihood of aggregation during this primary phase is unknown. Here, we use optical tweezers to measure the force between isolated *Bacillus subtilis* cells during approach. As we move the bacteria towards each other, cell motility (bacterial swimming) initiates the generation of repulsive forces at bacterial separations of ~3 μm. Moreover, the motile response displays spatial sensitivity with greater cell–cell repulsion evident as inter-bacterial distances decrease. To examine the environmental influence on the inter-bacterial forces, we perform the experiment with bacteria suspended in Tryptic Soy Broth, NaCl solution and deionised water. Our experiments demonstrate that repulsive forces are strongest in systems that inhibit biofilm formation (Tryptic Soy Broth), while attractive forces are weak and rare, even in systems where biofilms develop (NaCl solution). These results reveal that bacteria are able to control the likelihood of aggregation during the approach phase through a discretely modulated motile response. Clearly, the force-generating motility we observe during approach promotes biofilm prevention, rather than biofilm formation.

## Introduction

Biofilms are exploited in a wide range of biotechnologies, including wastewater treatment, biofuel production and the generation of electricity in microbial fuel cells [16, 21]. Conversely, however, they generate billions of dollars in losses each year through machinery damage, loss of processing and manufacturing efficiency, product contamination and medical infections [9, 12, 17, 22]. As a result, understanding the mechanisms of biofilm formation have become key to both the design of biofilm for optimal biotechnological use and the development of biofilm inhibitors preventing medical infections or equipment damage. Aggregation of planktonic cells, either to each other or to a biofilm, plays a key role in biofilm formation [1, 15, 18, 23]. Successful aggregation is driven on close contact between bacterial cells by physical forces such as attractive van der Waals and biological mechanisms, for example the bridging of protein adhesins and saccharide receptors between opposing cell walls [10, 15]. However, before these mechanisms can induce aggregation, bacteria must first approach each other either via swimming motility or through Brownian motion. While approach of planktonic cells is the critical first step in aggregation, we know nothing of how individual bacteria control this process.

**Fig. 1:**
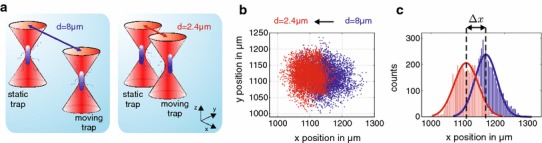
Optical force measurements between individual *Bacillus subtilis* bacteria. **a** Two optical traps hold a bacterium each at a distance varying between 2 and 8 μm. **b** For every separation distance *d* of each bacterial pair, one measurement records 50,000 positions of the bacterium in the *static trap*. **c** The force between the two bacteria is determined from the shift of the mean position Δ*x* of the bacterium in the *static trap* relative to its starting position at large separation distances (*d* = 6*–*8 μm) where no bacteria interaction is assumed

Here, we measured the force generated during approach of bacterial nearest neighbours using *Bacillus subtilis* cells trapped with optical tweezers (Fig. 1a). Although an optical trap restricts the bacterium’s position in space, its non-invasive nature leaves the bacterium’s natural motion unperturbed enabling its motile response to be analysed [2, 14, 19]. To measure the forces between two individual *Bacillus subtilis* cells, we held one cell in a fixed position (*static trap*) and tracked its motion, while we positioned a second cell (*moving trap*) within close vicinity. Recording the mean position of the bacterium in the *static trap* and monitoring its shift away from the trap centre, ∆*x* quantifies the external force induced by the bacterium in the *moving trap* (Fig. 1b, c). According to Hooke’s law *F* = −*κ *∆*x* (with the trap stiffness *κ*), we obtain a precise measurement of the force *F* exerted on the trapped bacterium in the *static trap*. To stimulate different levels of aggregation, we performed the experiments in three different media: Tryptic Soy broth (TSB), deionized water and 0.1 M NaCl solution. Deionized water and 0.1 M NaCl solution were chosen as they provided nutrient limited conditions, which promote biofilm formation in many bacteria, including *B. subtilis* [11, 25]. Indeed, we observed numerous aggregates of bacterial cells forming under 0.1 M NaCl solution, with occasional bacterial aggregates occurring in deionized water and none for cells suspended in TSB.

## Materials and Methods

### Holographic Optical Tweezers

**Fig. 2:**
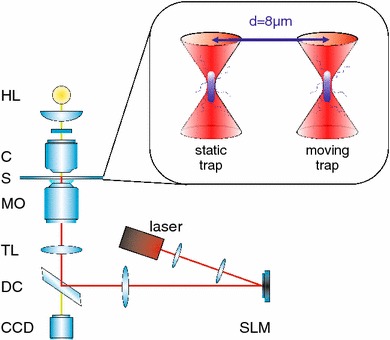
Schematic of the experimental setup. A Ti:Sapphire laser (M Squared, SolsTiS, 1.5 W, 790–850 nm) is split into two traps by a spatial light modulator (SLM, Boulder Nonlinear Systems, XY Series, 512 × 512 pixels). After passing a dichroic mirror (*DC*) and the tube lens (*TL*), the laser enters the microscope objective (*MO*, Nikon CFI Plan Fluor, oil immersion, NA 1.3) and is focussed into the sample chamber (S), which contains the bacteria solution sandwiched between two glass coverslips. A halogen bulb (*HL*) illuminates the sample through the condenser (C, Zeiss NA 0.9), and a* CCD* camera (Prosilica GE680C) records the images of the bacteria in the traps. A sketch of the trap setup inside the sample cell is displayed in the *inset*

Figure 2 shows a schematic diagram of the inverted optical tweezers setup, and an extensive description is detailed elsewhere [13]. In essence, we split a Ti:Sapphire laser beam (830 nm) with a spatial light modulator (SLM, Boulder Nonlinear Systems) and focussed it tightly with a 100 × Nikon microscope objective (NA 1.3) into the sample chamber. The SLM controls the two generated optical traps independently, and they are positioned 10 μm deep in the sample to avoid any interaction of the bacteria with the glass cover slip. We kept the laser power in each trap very low (3–5 mW) to avoid damaging the bacteria and to provide as much possible freedom for a motile response. Trapping bacteria at low power in a weak trap results in a low trap stiffness, which in turn enables us to measure very small forces. The bacteria actively swam away from the trap site once we turned off the trapping laser, which indicates good bacterial viability even after longer periods of trapping. We performed all experiments at room temperature (22 °C).

### Data Acquisition and Force Measurements

We keep the first trap at the same position at all times (*static trap*), whereas we move the second trap laterally with respect to the first in a step-wise fashion. During position measurement, both traps remain stationary. In order to avoid an overlap of the optical trapping potentials, we did not move the traps closer than 2 μm. The bacteria's average diameter is 500 nm, which leaves approximately 1 μm between cell walls at the closest separation. For each set distance between the *static* and the *moving trap*, we take 50,000 position measurements of the bacterium in the* static trap* along its short axis. We continue by positioning the *moving trap* closer to the *static trap* in 100 nm steps, taking a measurement every 500 nm and for separation distances below 3 μm every 200 nm. Once the bacteria are 2 μm apart we separate them again, tracing back the positions of the *moving trap* and repeat measuring the position of the bacteria in the *static trap* every 200/500 nm as on approach. The position measurements at each separation distance take 25 s to complete. Overall, measuring the positions of one bacterial pair as well as moving the traps close together and separating them again last for 20 min.

We apply Hooke’s law to obtain the force from the displacement ∆*x* of the mean position of the bacteria in the trap: *F* = −*κx*∆ with the trap stiffness *κ* = *k*_B_ *T*/(*σ*_x_)^2^. Here, *k*_B_ is Boltzmann’s constant, the temperature is *T* = *295.15* *K* and *σ*_x_ is the standard deviation of the measured position data. We measure the trap stiffness *κ* for every separation distance and average all the obtained values for *κ* over one bacterial pair. The mean position of a trapped 1 μm polystyrene bead in our control experiment varies between ±3 nm, which is within the experimental error limit. We track the trapped bacteria’s motion by recording the video images for the particle tracking analysis with a high-speed camera (Prosilica GE680C) at 2,000 Hz. We control all devices as well as the image acquisition and particle tracking with customised LabVIEW software (National Instruments) created by Bowman [6].

### Bacteria Sample Preparation

*Bacillus subtilis* cultures (ATCC 23,857) were grown in Tryptic Soy Broth at 30 °C on a rotary shaker at 130 rpm for ~18 h. We centrifuged 0.5 ml of culture for 2 min at 1,000 rpm and subsequently removed the supernatant media. The remaining bacteria were transferred into new media (either TSB, deionized water or 0.1 M NaCl solution). Using a plastic pipette, we applied the bacteria in the new media to a single concave microscopic slide and sealed the cover slip to the slide with petroleum jelly.

## Results

**Fig. 3:**
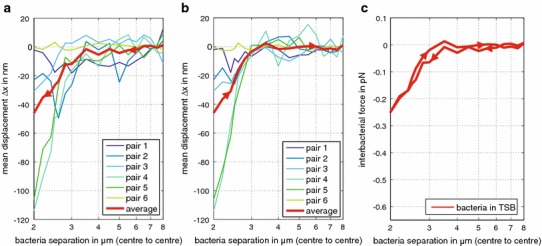
Mean displacements and inter-bacterial forces between bacterial pairs in Tryptic Soy Broth (TSB). **a** The mean displacement Δ*x* of the bacterium in the *static trap* from the trap centre increases on approach of the second bacterium in the *moving trap*. Results are shown for six separate bacterial pairs and their combined average. **b** The mean displacement Δ*x* of the bacterium in the *static trap* from the trap centre on retreat of the second bacterium closely follows the trend on approach. **c** Summary of average inter-bacterial forces calculated from Δ*x* on approach and retreat. All measurements are corrected for drift

**Fig. 4:**
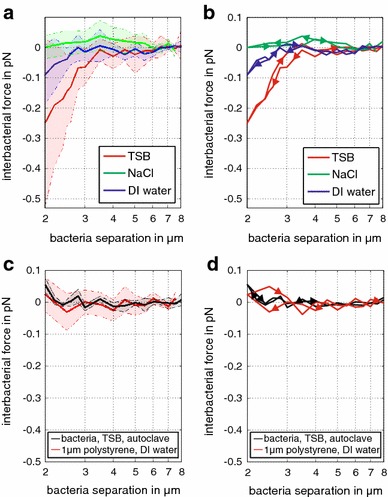
Environmental influence on inter-bacterial forces. **a** The repulsive force (each *force line* represents the average of six bacterial pairs) on approach is strongest for bacteria suspended in TSB, decreases for nutrient-deprived deionized water and disappears for bacteria in NaCl solution. The* shaded areas* represent the standard deviation of the force at each separation distance for the six bacterial pairs. **b** Averaged *force lines* follow the same trend on approach and retreat. **c**–**d** Average force and standard deviation on approach and retreat for autoclaved bacteria in TSB and 1 μm polystyrene beads in deionized water show no repulsion, indicating that we observe a biological response in living samples

**Fig. 5:**
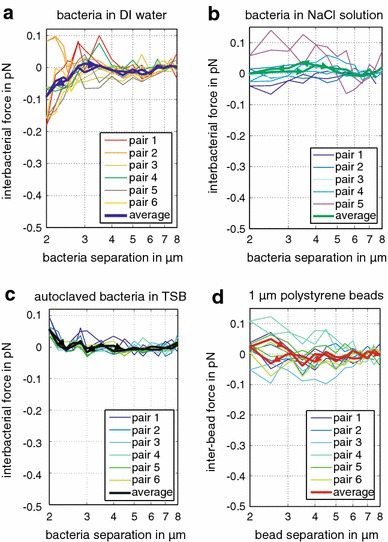
Environmental influence on inter-bacterial forces. The graphs display the complete datasets for the average *force lines* displayed in Fig. 4. Force on approach and retreat for bacteria suspended in **a** deionized water, **b** NaCl solution, **c** autoclaved bacteria in TSB and **d** 1 μm polystyrene beads. Each* bold line* represents the force average of six bacterial pairs or six polystyrene bead pairs. Only the interaction of bacteria in deionized water result in a repulsive force for trap separations below 3 μm. All other experiments displayed no significant change in force for decreasing the separation distance of the traps. All measurements are corrected for drift

The first set of experiments investigated *B. subtilis* cells suspended in TSB. As we decreased the separation distance *d* between bacteria, a majority of bacteria in the *static trap* began to show repulsion (they repelled themselves from their neighbour in the *moving trap*) at a centre-to-centre distance of approximately 3.5 µm (Fig. 3a, c). Intriguingly, as the distance between the bacteria decreased, cells commonly showed an increase in repulsive force, reaching an average maximum of 0.25 pN at a centre-to-centre separation of 2 µm (Fig. 3a, c). Then, as we moved the cells apart, the repulsive force decreased, following a similar trend to that observed during cell–cell approach (Fig. 3b, c). We repeated the experiment with cells suspended in either 0.1 M NaCl solution or deionized water (Figs. 4a, b, 5). In deionised water, again, a majority of cells displayed an increase in repulsive force as they moved closer, but the maximum average repulsive force (0.09 pN) was considerably weaker than in TSB (0.25 pN). As before, the repulsive force decreased as the optical traps moved the cells apart, closely tracking the trend observed on approach (Figs. 4b, 5a). We measured no significant repulsive force undertaking identical experiments in 0.1 M NaCl solution (Figs. 4a, b, 5b).

Overall, our results show that the strongest repulsive forces between two individual bacteria occur under nutrient-rich conditions, which did not promote aggregation. Conversely, weaker or absent repulsive forces dominated in systems where aggregation occurred (deionized water or 0.1 M NaCl solution). Thus, we suggest the strong cell–cell repulsion seen on approach plays a role in preventing aggregation in the nutrient-rich TSB, while the weaker or absent repulsive forces facilitate aggregation in the nutrient-starved systems. Notably, significant attractive forces were rare and did not show systematic trends. Evidently, attractive forces are not required on approach to promote aggregation. Instead, when forces are generated on approach, they are repulsive in nature and dedicated to prevent aggregation.

We argue that the repulsive forces observed must result from cell motility (bacterial swimming). We can categorically exclude electrostatic repulsion, because these forces only extend tens of nanometres from the cell surface [17]; thus, cannot explain the repulsion we saw at separations of microns. While a majority of cells in TSB and deionized water displayed motile repulsion, there was significant variability in the motile force generated by different bacterial pairs. This was most notable in TSB where forces ranging from zero to 0.62 pN at the closest separations. Clearly, even in TSB, some cells displayed significant repulsion while others displayed none. However, *B. subtilis* cells from a single culture are known to display intracellular variability in the extent of motility [5]; thus, we argue that significant variations in the extent of the repulsive force must be expected.

As a control, we repeated the experiments in nutrient-rich TSB media with deactivated (autoclaved) bacteria, which were killed to prevent motility. We observed no repulsion (Fig. 4c, d), supporting the hypothesis that motility appears to be responsible for repulsion observed for functioning bacteria. Conspicuously, a small attractive force of 0.04 pN was observed at the closest separation. However, our control experiments with 1 μm polystyrene beads showed that this small attractive force was within the force variability displayed by the beads (Fig. 4c, d). As a comparison, the repulsive forces we measured for functioning bacteria in TSB and deionized water exceed at least twice the magnitude of the force variability displayed by polystyrene beads and autoclaved bacteria.

## Discussion

Motility is known to both aid and inhibit biofilm formation. For example, examination of dual species biofilms containing *Pseudomonas Aeruginosa* and *Agrobacterium tumefaciens* reveals that *A. tumefaciens* cells use motility as an escape mechanism to prevent incorporation into the biofilm [3]. *A. tumefaciens* cells, however, were already a part of the biofilm. Our work here reveals that motile escape mechanisms can exist prior to aggregation, thus inhibiting the earliest steps of biofilm formation. Equally, upregulation of flagella synthesis and motility genes has been shown to stimulate biofilm formation; the upregulation was directed by quorum sensing [4]. Quorum sensing enables bacteria to detect the relative density of their surrounding bacterial population via the production of autoinducer molecules. As these accumulate within the cell, upregulation of specific genes occurs above certain concentration thresholds, inducing a wide variety of responses, including biofilm formation [24, 26]. Most importantly, quorum sensing is a community-wide response—a response to the bulk density of the surrounding population. It has not yet been shown to facilitate communication between two individual bacteria, nor their spatial awareness of each other.

In our study, the close relationship between separation distance and force suggests a spatial sensitivity between bacterial pairs and a form of communication between the two cells. Our experiments, however, do not reveal the mechanism by which communication occurs. One may speculate that the bacteria are sensing a concentration gradient of a compound excreted by the organism, and thus the motile repulsion is a chemotactic response. Indeed, the ability to influence motility through sensing concentration gradients of excreted compounds has been shown for *Escherichia coli* [7, 20], opening the possibility that an analogous mechanism may be utilised by *B. subtilis* to prevent aggregation. Alternatively, inter-bacterial sensing may result from contact of the flagella of the nearest neighbour. Indeed, *B. subtilus* cells can display flagella over 3 µm long [8], and therefore flagella contact is possible at the distances where we observed cell–cell repulsion (<3 µm). However, it is unlikely that the physical presence of flagella alone is causing repulsion simply by behaving as a spring mechanism. If this was the case, we would expect to see equal repulsion in all three liquid media. Whatever mechanism is utilised to sense inter-bacterial distances, it is evident that the bacteria are able to adjust their motile response to control the likelihood of aggregation. Importantly, these are individual responses of nearest neighbours, which act in isolation from the rest of the community. Yet overall, the combined response of individuals leads to the global phenomenon of either biofilm formation or prevention.

## References

[1] Al-Bakri AG, Gilbert P, Allison DG (2004). Immigration and emigration of* Burkholderia cepacia* and* Pseudomonas aeruginosa* between and within mixed biofilm communities. J Appl Microbiol.

[2] Altindal T, Chattopadhyay S, Wu X (2011). Bacterial chemotaxis in an optical trap. PLoS ONE.

[3] An D, Danhorn T, Fuqua C, Parsek MR (2006). Quorum sensing and motility mediate interactions between* Pseudomonas aeruginosa* and* Agrobacterium tumefaciens* in biofilm cocultures. PNAS.

[4] Barrios AFG, Zuo R, Hashimoto Y, Yang L, Bentley WE, Wood TK (2006). Autoinducer 2 controls biofilm formation in* Escherichia coli* through a novel motility quorum-sensing regulator (MqsR, B3022). J Bacteriol.

[5] Blair KM, Turner L, Winkelman JT, Berg HC, Kearns DB (2008). A molecular clutch disables flagella in the *Bacillus subtilis* biofilm. Science.

[6] Bowman R (2012) Optical tweezers Software, www.gla.ac.uk/schools/physics/research/groups/optics/research/opticaltweezers/software

[7] Budrene EO, Berg HC (1991). Complex patterns formed by motile cells of* Escherichia coli*. Nature.

[8] Cisneros LH, Cortez R, Dombrowski C, Goldstein RE, Kessler JO (2007). Fluid dynamics of self-propelled microorganisms, from individuals to concentrated populations. Exp Fluids.

[9] Coraca-Huber DC, Fille M, Hausdorfer J, Pfaller K, Nogler M (2012). *Staphylococcus aureus *biofilm formation and antibiotic susceptibility tests on polystyrene and metal surfaces. J Appl Microbiol.

[10] Das T, Krom BP, van der Mei HC, Busscher HJ, Sharma PK (2011). DNA-mediated bacterial aggregation is dictated by acid–base interactions. Soft Matter.

[11] Elhariry HM (2008). Biofilm formation by endospore-forming bacilli on plastic surface under some food-related and environmental stress conditions. Global J Biotechnol Biochem.

[12] Flemming HC (2011). Microbial Biofouling: unsolved problems, unsufficient approaches and possible solutions. Biofilm Highlights.

[13] Gibson GM, Leach J, Keen S, Wright AJ, Padgett MJ (2008). Measuring the accuracy of particle position and force in optical tweezers using high-speed video microscopy. Opt Express.

[14] Kemper B, Barroso A, Woerdemann M, Dewenter L, Vollmer A, Schubert R, Mellmann A, von Bally G, Denz c (2013). Towards 3D modelling and imaging of infection scenarios at the single cell level using holographic optical tweezers and digital holographic microscopy. J Biophotonics.

[15] Kolenbrander PE, Palmer RJ, Periasamy S, Jakubovics NS (2010). Oral multispecies biofilm development and the key role of cell–cell distance. Nat Rev Microbiol.

[16] Logan BE, Hamelers B, Rozendal R, Schröder U, Keller J, Freguia S, Aelterman P, Verstraete W, Rabaey K (2006). Microbial fuel cells: methodology and technology. Environ Sci Technol.

[17] Lynch AS, Robertson GT (2008). Bacterial and fungal biofilm infections. Annu Rev Med.

[18] Malik A, Sakamoto M, Hanazaki S, Osawa M, Suzuki T, Tochigi M, Kakii K (2003). Coaggregation among nonflocculating bacteria isolated from activated sludge. Appl Environ Microbiol.

[19] Min TL, Mears PJ, Chubiz LM, Rao CV, Golding I, Chemla YR (2009). High-resolution, long-term characterization of bacterial motility using optical tweezers. Nat Methods.

[20] Mittal N, Budrene EO, Brenner MP, van Oudenaarden A (2003). Motility of* Escherichia coli* cells in clusters formed by chemotactic aggregation. PNAS.

[21] Qureshi N, Annous BA, Ezeji TC, Karcher P, Maddox IS (2005). Biofilm reactors for industrial bioconversion processes: employing potential of enhanced reaction rates. Microb Cell Fact.

[22] Ren D, Sims JJ, Wood TK (2002). Inhibition of biofilm formation and swarming of* Bacillus subtilis* by (5Z)-4-bromo-5-(bromomethylene)- 3-butyl-2(5H)-furanone. Lett Appl Microbiol.

[23] Rickard AH, Gilbert P, High NJ, Kolenbrander PE, Handley PS (2003). Bacterial coaggregation: an integral process in the development of multi-species biofilms. Trends Microbiol.

[24] Smith RS, Iglewski BH (2003). *P. aeruginosa* quorum-sensing systems and virulence. Curr Opin Microbiol.

[25] Stanley NR, Britton RA, Grossman AD, Lazazzera BA (2003). Identification of catabolite repression as a physiological regulator of biofilm formation by* Bacillus subtilis* by Use of DNA Microarrays. J Bacteriol.

[26] Waters CM, Bassler BL (2005). Quorum sensing: cell-to-cell communication in bacteria. Annu Rev Cell Dev Bi.

